# Nursing training and retraining on cardiopulmonary resuscitation: a theoretical-practical intervention*

**DOI:** 10.1590/1980-220X-REEUSP-2021-0521

**Published:** 2022-04-27

**Authors:** Nyagra Ribeiro de Araujo, Raul Amaral de Araújo, Miguel Antonio Moretti, Antonio Carlos Palandri Chagas

**Affiliations:** 1 Centro Universitário FMABC, Faculdade de Medicina do ABC, Programa de Pós-Graduação em Ciências da Saúde, Santo André, SP, Brazil.; 2 Universidade Federal de Pernambuco, Departamento de Prótese e Cirugia Bucofacial, Recife, PE, Brazil.; 3 Centro Universitário FMABC, Faculdade de Medicina do ABC, Departamento de Cardiologia, Santo André, SP, Brazil.

**Keywords:** Nurse Practitioners, Education, Continuing, Inservice Training, Cardiopulmonary Resuscitation, Knowledge, Motor Skills, Profissionais de Enfermagem, Educação Continuada, Capacitação em Serviço, Reanimação Cardiopulmonar, Conhecimento, Destreza Motora, Profissionais de Enfermagem, Educação Continuada, Capacitação em Serviço, Reanimação Cardiopulmonar, Conhecimento, Destreza Motora

## Abstract

**Objective:**

To analyze the retention of knowledge and skills of nursing professionals following training and retraining on cardiopulmonary resuscitation.

**Method:**

This is an intervention, prospective, and analytical study in which 56 nursing professionals received theoretical and practical training in in-service cardiopulmonary resuscitation. Nine months after the first training (T1), these professionals participated in a retraining (T2). They were followed up for 18 months. The linear trend of knowledge and skills in the period following training was calculated and the Wilcoxon test was applied.

**Results:**

Interventions increased the knowledge and skills of professionals significantly; however, in the subsequent period, skills decreased. Despite this, after a period of nine months, they were still higher than those identified before the study. There was a reduction of 18.2% in knowledge in the theoretical test after T1 vs 13.0% after T2 (p < 0.01) and a reduction of 7.6% in skills on the practical test after T1 vs 5.3% after T2 (p < 0.01).

**Conclusion:**

Nurses were able to retain more knowledge and skills on cardiopulmonary resuscitation after retraining, which stresses the importance of regular training and continuing education in health.

## RESUMO

**Objetivo:**

Analisar a retenção do conhecimento e habilidades dos profissionais de enfermagem após treinamento e retreinamento sobre ressuscitação cardiopulmonar.

**Método:**

Estudo de intervenção, prospectivo e analítico no qual 56 profissionais de enfermagem receberam treinamento teórico e prático de RCP em serviço. Nove meses após o primeiro treinamento (T1), esses profissionais participaram de um retreinamento (T2). Eles foram acompanhados por 18 meses. A tendência linear do conhecimento e habilidades no período subsequente aos treinamentos foi calculada e aplicou-se o teste de Wilcoxon.

**Resultados:**

As intervenções aumentaram significativamente o conhecimento e as habilidades dos profissionais; no entanto, no período subsequente, as competências reduziram. Apesar disso, após um período de nove meses elas ainda se mantiveram superiores àquelas identificadas previamente ao estudo. Houve redução de 18,2% após T1 vs 13,0% após T2 (p < 0,01) do conhecimento no teste teórico e de 7,6% após T1 vs 5,3% após T2 (p < 0,01) das habilidades no teste prático.

**Conclusáo:**

Os profissionais de enfermagem conseguiram reter mais conhecimento e habilidades em ressuscitação cardiopulmonar após o retreinamento, o que reforça a importância da regularidade das capacitações e da educação continuada em saúde.

## RESUMEN

**Objetivo:**

Analizar la retención del conocimiento y habilidades de los profesionales de enfermería tras entrenamiento y reentrenamiento sobre reanimación cardiopulmonar (RCP).

**Método:**

Estudio de intervención, prospectivo y analítico en el cual 56 profesionales de enfermería recibieron entrenamiento teórico y práctico de RCP en servicio. Nueve meses tras el primer entrenamiento (E1), esos profesionales participaron de un reentrenamiento (E2). Ellos fueron acompanados por 18 meses. La tendencia lineal del conocimiento y habilidades en el período subsecuente a los entrenamientos fue calculada y se aplicó el test de Wilcoxon.

**Resultados:**

Las intervenciones aumentaron significativamente el conocimiento y las habilidades de los profesionales, sin embargo, en el periodo subsecuente las competencias se redujeron. A pesar de ello, tras un período de nueve meses ellas todavía se mantuvieron superiores a aquellas identificadas previamente al estudio. Hubo reducción del 18,2% tras E1 contra el 13,0% tras E2 (p < 0,01) del conocimiento en el test teórico y del 7,6% tras E1 contra el 5,3% tras E2 (p < 0,01) de las habilidades en el test práctico.

**Conclusión:**

Los profesionales de enfermería lograron retener más conocimiento y habilidades en RCP tras el reentrenamiento, lo que refuerza la importancia de la regularidad de cursos de capacitación y de la educación continua en salud.

## INTRODUCTION

There is considerable variability in survival rates after cardiopulmonary arrest (CPA) that cannot be attributed solely to the patient's characteristics. Good results are related to early initiation of high-quality cardiopulmonary resuscitation (CPR), rapid defibrillation, qualified staff and an organizational structure that supports care after resuscitation^(1)^.

CPR comprises procedures for victims presenting signs of CPA (lack of verbal response, breathing and palpable pulse) and has as main components chest compressions and ventilations. In the hospital setting, the nursing team is usually the first to identify CPA situations and start resuscitation maneuvers; they also activate the emergency call, provide the necessary supplies and act as an articulator between the other team members, thus providing an agile, synchronized and efficient service, maximizing quality of care^(2)^. Despite this, there are gaps in the knowledge and practical skills of these professionals in performing high-quality CPR that can interfere with patient outcomes and chances of survival after cardiac arrest. Thus, continuing education on the subject is important^(3)^.

CPR training increases the professional's knowledge immediately after its completion; however, a decline in theoretical and practical skills acquired over time is also observed if they are poorly performed or trained^(4)^. There is no exact moment from which the retention of theoretical and practical knowledge declines, and although better performance is observed in individuals who train more regularly, the ideal time interval between trainings remains unknown^(5)^.

Prolonged intervals greater than one or two years seemed insufficient to retain CPR skills^(6)^ and, although the results of some intervals lower than this period are documented in the literature, the clinical and methodological heterogeneity of the studies makes it difficult to reach a consensus on this ideal interval^(7)^. Another gap is the lack of studies with methodologies and training intervals that can guide continuing education in service, especially the one involving nursing professionals, a category with exclusive and relevant attributions in in-hospital CPA care.

Hospitals that excelled in in-hospital CPA survival emphasized nursing guidance and training and ensured clinical competence and adequate nursing training for care in CPR situations^(8)^. Therefore, developing studies that investigate adequate intervals and in-service training methodologies that improve the retention of skills in resuscitation is essential to equip health institutions in the process of continuing education in CPR for nursing professionals, aiming better quality of the care provided. Therefore, this study aimed at analyzing the retention of knowledge and skills of nursing professionals following training and retraining on cardiopulmonary resuscitation.

## METHOD

### Design of Study and Local

Intervention, prospective, and analytical study carried out at the Hospital Regional do Cariri (HRC) located in Juazeiro do Norte-CE. This is the first public, tertiary and large hospital of the state network built in the inland region of Ceará, reference for the care of the population of Cariri's 44 municipalities in its health macro-region, mainly in situations of trauma and cerebrovascular accident. This institution is certified as Accredited with Excellence - Level 3, from the National Accreditation Organization (ONA). It develops Continuing Education actions for its employees through the Teaching and Research Sector on various topics; however, CPR training takes place on a timely manner, without periodicity and specific methodology. Thus, this study also aimed to help this institution in the planning and continuity of its CPR education actions for nursing professionals, something already recognized as relevant and necessary.

### Population and Selection Criteria

The study population consisted of nurses and nursing technicians who performed their activities in a clinical care unit in a ward or in an intensive care unit of the aforementioned institution. Professionals who had worked at the institution for at least six months were included and those who did not participate in all stages of the study were excluded.

### Sample Definition

For sample delimitation, sample size calculation was performed through the sample size calculation equation to study the mean in paired samples, using the following parameters: significance level of 5%, test power of 85% and increase of 2 points in knowledge and skills of professionals (20%) with a variance of 9 points before and after the intervention. This calculation defined a minimum number of 41 professionals to be observed at each evaluation moment. Due to the follow-up time and the employee turnover profile at the institution, the study started with twice the minimum sample, 82 professionals.

They were selected randomly and proportionally in relation to their training (nurse and nursing technician) and the sector of the institution in which they worked (internal medicine, surgical clinic, trauma-orthopedic clinic, special care unit, stroke unit, and intensive care unit).

### Intervention

The educational intervention carried out corresponded to two short-term theoretical and practical training sessions in CPR with a focus on Basic Life Support (BLS) and some elements of Advanced Cardiology Life Support (ACLS) at the theoretical moment, since the professionals work in hospital settings, and emphasis on BLS in the practical moment, to prioritize cardiac arrest recognition and CPR maneuvers.

They lasted an average of one hour and thirty minutes, were performed in-service, and were taught by an American Heart Association (AHA) BLS course-certified instructor. Participants were divided into groups of a maximum of six people for better use of practical activities. In the theoretical moment, the following contents were addressed: definition and rhythms of CPA, chain of survival in an in-hospital and extra-hospital CPA, adult CPA algorithm, chest compression, ventilation technique with barrier devices and bag-valve mask, use of automatic external defibrillator (AED) and ACLS basics (use of manual defibrillator, ventilation with advanced airway and medications used in CPA); in the practical moment, recognition of CPA, technique of chest compressions and ventilation, use of AED and the sequence of BLS with one and two rescuers. The training time was shorter than the standard AHA BLS course as only content related to adult CPA was taught.

The teaching strategy used was the approach of theoretical aspects with immediate practical demonstration of all actions and maneuvers. Lectures and multimedia resources with images and animations were adopted in the CPA care and at the end a practical demonstration of the complete sequence of a care session was carried out, being performed according to the AHA's BLS course. It should be noted that the participants trained all the maneuvers and techniques demonstrated in the educational activity.

### Data Collection

Data were collected from April/2018 to March/2020. To this end, two instruments were used: a) a questionnaire with 25 multiple choice questions about the subjects taught at the theoretical moment of the training, which allowed the assessment of knowledge in CPR; b) checklist with 18 items that allowed assessing practical skills in CPR while the participant simulated care for a patient in cardiac arrest, on a low-fidelity manikin, with a bag-valve-mask device and training AED. At this moment, the professional should recognize CPA, correctly use the available materials (bag-valve mask and AED), correctly perform the ventilation and compression techniques and complete at least one CPR cycle (30:2). The instruments were structured and applied similarly to the tests used in the AHA BLS courses and developed according to the 2015 CPR guidelines, having been previously analyzed by five experts. The score of both corresponded to the absolute number of correct answers of the evaluated items.

**Figure 1:**
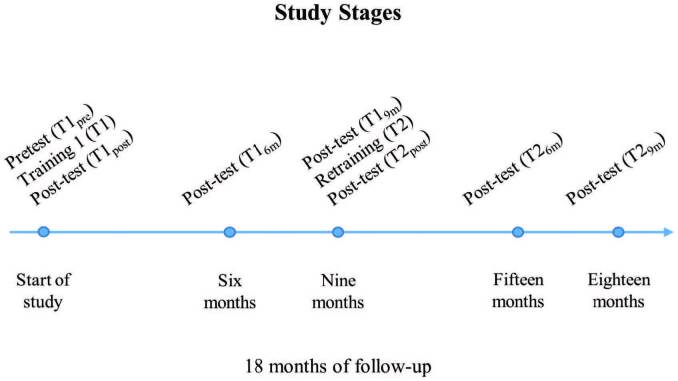
Study flowchart – Juazeiro do Norte, CE, Brazil, 2020.

Before the first training (T1_pre_) each participant took the theoretical and practical test to identify pre-existing knowledge regarding CPR. Immediately after the first training (T1_post_), the professionals underwent the same theoretical and practical tests to assess their learning after the intervention. After a period, during which there was no training, they were subjected to new tests in the next sixth (T1_6m_) and ninth month (T1_9m_) to assess how the retention of knowledge and skills in CPR behaved over that time. Nine months after the first training (T1), these professionals underwent retraining (T2), with format and methodology similar to the previous one. Immediately after T2 (T2_post_) the professionals underwent the theoretical and practical tests again, as well as in the sixth (T2_6m_) and ninth month (T2_9m_), for a total of 18 months of follow-up (Figure 1).

The nine-month interval between trainings was defined as this is a period whose effect is not documented in the literature and, in view of the reduction in CPR skills that occurs in periods of less than one year^(7)^, other intervals have to be investigated. Assessment at six months was performed to identify whether there would be a reduction in theoretical and practical knowledge that would indicate the need or not for earlier retraining.

The theoretical knowledge assessment instrument had the same content in all its applications, being modified only in the items that refer to the enumeration, writing of the questions and formatting, to mask the test for the participant. The skills assessment instrument also maintained the same content and was applied by the same evaluator at all times, to minimize interpretation bias. This allowed the assessment of the same CPR competencies throughout the research execution.

### Data Analysis and Treatment

Data were stored in a database and analyzed using the software Statistical Package for the Social Sciences (SPSS), version 20.0. The profile of the professionals was described in a table and the values expressed in absolute and relative numbers. To analyze the knowledge and skills in CPR at each moment of the study, the absolute number of correct answers in each theoretical (25 items) and practical (18 items) assessment was used, with the values expressed as mean and standard deviation in a line graph. The linear trend of the reduction of knowledge and skills was calculated in the two periods following the training, which was expressed in percentage numbers, with the first period being T1_post_ until T1_9m_ and the second of T2_post_ until T2_9m_.

The assessment of the normality of the knowledge and skills score was performed using the Shapiro-Wilk test, which indicated a non-normal distribution. The comparison of the evolution of knowledge and skills between the different moments was performed using the Wilcoxon test. The confidence level was 95% and the statistical significance considered for p < 0.05.

### Ethical Aspects

The research project was approved by the Research Ethics Committee of the Institute of Health and Hospital Management (ISGH), under Opinion No. 2.396.486, 2017, following the recommendations of Resolution 466/12, of the National Health Council, which regulates research with human beings. All participants signed the Free and Informed Consent Form.

## RESULTS

After follow-up losses, the analyzed sample consisted of 56 nursing professionals distributed as follows: female (82.1%), aged 19 to 30 years and 31 to 40 years (both with 37.5%), nursing technician profession (62.5%), and training time between 6 and 10 years (46.4%). Most had worked for two years or less at the institution (44.6%), worked in only one place (64.7%), performed their activities in a clinical care unit in a ward (60.7%), had the last training in CPR more than 1 year before (64.3%), and worked with high frequency in CPR situations (66.1%) (Table 1).

**Table 1:** Characterization of nursing professionals (n = 56) – Juazeiro do Norte, CE, Brazil, 2020.

Variables	n	(%)
**Sex**		
Female	46	(82.1)
Male	10	(17.9)
**Age**		
19 to 30 years	21	(37.5)
31 to 40 years	21	(37.5)
Over 40 years	14	(25.0)
**Occupation**		
Nurse	21	(37.5)
Nursing technician	35	(62.5)
**Time since graduation**		
Up to 5 years	11	(19.6)
6 to 10 years	26	(46.4)
Over 10 years	19	(33.9)
**Length of time working at the current institution**		
Up to 2 years	25	(44.6)
Over 2 to 5 years	22	(39.3)
Over 5 years	9	(16.1)
**Another workplace**		
Yes	20	(35.7)
No	36	(64.7)
**Time of last CPR training**		
Equal to or less than 1 year	20	(35.7)
Over 1 year	36	(64.3)
**Work unit**		
Clinical ward care	34	(60.7)
Intensive care	22	(39.3)
**Frequency of work in CPR situations**		
Low (<1 CPR per month)	19	(33.9)
High (>1 CPR per month)	37	(66.1)

Note: CPR: Cardiopulmonary resuscitation.

**Figure 2:**
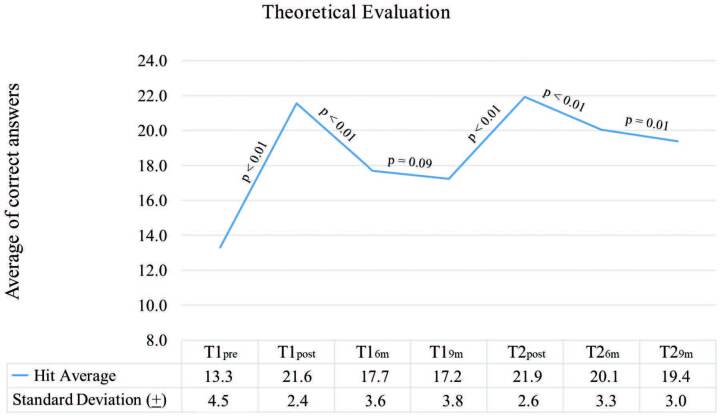
Evolution of the theoretical knowledge of nursing professionals according to the moment of evaluation – Juazeiro do Norte, CE, Brazil, 2020.

Note: T1_pre_: pre-test of training 1; T1_post_: post-test of training 1; T1_6m_: six months after training 1; T1_9m_: nine months after training 1; T2_post_: post-test of training 2; T2_6m_: six months after training 2; T2_9m_: nine months after training 2; p-value of the Wilcoxon test.

The average of correct answers before the intervention was 13.3 (±4.5) in the theoretical test. Immediately after training (T1 and T2), there was a significant increase in knowledge and the average of correct answers in the post-test of these two moments were, respectively, 21.6 (±2.4) and 21.9 (±2.6), p = 0.12. There was no significant reduction in the period of T1_6m_ to T1_9m_ (p = 0.09). The average of correct answers in the theoretical evaluation performed nine months after each training, despite having a drop in both periods, was higher after retraining (T1_9m_= 17.2 (±3.8) vs T2_9m_= 19.4 (±3.0), p < 0.01). The evolution of average of correct answers in the theoretical test can be seen in Figure 2.

**Figure 3:**
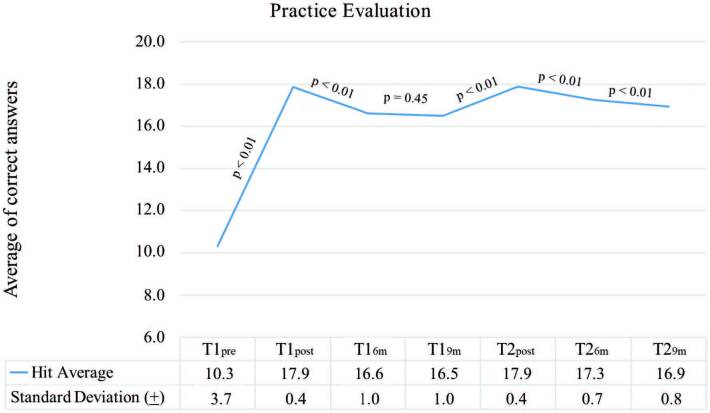
Evolution of practical skills of nursing professionals according to the moment of evaluation – Juazeiro do Norte, CE, Brazil, 2020.

Note: T1_pre_: pre-test of training 1; T1_post_: post-test of training 1; T1_6m_: six months after training 1; T1_9m_: nine months after training 1; T2_post_: post-test of training 2; T2_6m_: six months after training 2; T2_9m_: nine months after training 2; p-value of the Wilcoxon test.

Regarding practical skills (Figure 3), the average of correct answers before the intervention was 10.3 (±3.7). The evolution of these skills after training was similar to that of knowledge, there was also no significant reduction in the period from T1_6m_ to T1_9m_ (p = 0.45). Immediately after T1 and T2, there was a significant increase in skills and the average of correct answers in the post-test of these two moments were statistically equal (17.9 (±0.4) vs 17.9 (±0.4), p = 0, 56); however, the average of correct answers was different and statistically higher nine months after the second training (T1_9m_ = 16.5 (±1.0) vs T2_9m_ = 16.9 (±0.8), p < 0.01).

**Figure 4:**
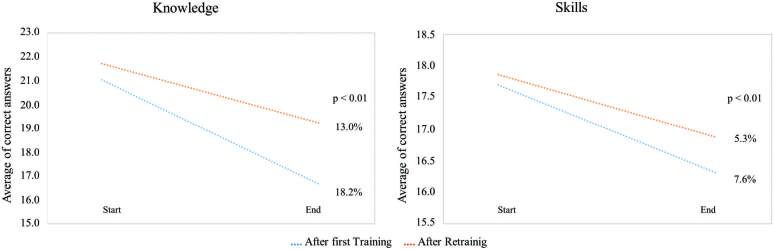
Comparison of the linear trend of percentage reduction in knowledge and skills of nursing professionals after the first training and retraining – Juazeiro do Norte, CE, Brazil, 2020.

Note: p-value of the Wilcoxon test.

In Figure 4, it is observed that after both theoretical and practical training there was a reduction in CPR competences and that it was significantly greater after the first training (T1_post_ to T1_9m_) than in retraining (T2_post_ to T2_9m_). Theoretical knowledge reduced 18.2% after T1 and 13.0% after T2, p < 0.01, and skills 7.6% and 5.3%, p < 0.01. Thus, retraining after nine months resulted in a higher average of correct answers and greater retention of theory and practice, despite the learning being similar at T1 and T2.

## DISCUSSION

The present study emphasizes that theoretical-practical trainings in CPR are fundamental strategies to retain and improve the skills of nursing professionals and highlights the importance of regular training, because if new educational activities are not carried out or are carried out in a timely manner, professionals' skills tend to reduce over time to levels that are insufficient for adequate care in a real emergency situation.

Regarding the professionals' profile, it was observed that most had their last training in CPR more than a year before, despite frequently working in CPR situations, which reinforces the continuity of research on the subject, especially involving strategies of in-service education. Among the factors that may interfere in this process of training nursing professionals are the lack of time, financial resources, nearby training centers, the lack of requirement of CPR training in the workplace, and also the lack of interest on the part of the professional^(9)^.

As typical rescuers in cardiac arrest, nurses and other healthcare professionals need to have adequate resuscitation skills^(10)^. However, before the first training, insufficient theoretical knowledge and motor skills was observed, in view of the average of correct answers in the tests. Although they are usually the first to identify situations of in-hospital CPA, nursing professionals still have difficulties in relation to the recognition of cardiac arrest, initial procedures, BLS, medications used, and drug administration routes^(3,11)^.

It is known that in-hospital CPA care is carried out as a team, together with the participation of other professionals, who, like the nurses, also have some deficit in theoretical and practical knowledge in CPR^(12)^. Among the reasons for the service provided by the teams being below the desirable level, the difficulty of maintaining acquired knowledge is considered; therefore, developing theoretical and practical training for all health professionals and establishing a periodicity of training are crucial measures^(13)^.

After training, there was a significant increase in knowledge and skills and a final score of correct answers above 84%, as expected (and suggested) in AHA training, a world reference in CPR training. In a similar way, research has shown an increase of 20.4% to 20.9%^(14)^, 28%^(11)^, 45%^(15)^, in theoretical knowledge after training. Regarding skills, there was a percentage of correct answers of 81.5%^(16)^ and up to 95%^(15)^ immediately after the educational intervention.

Training, especially those involving theoretical and practical activities, are the most effective and relevant means to increase and retain knowledge and skills in CPR. It should be highlighted that continuing education courses on the subject also promote updating, allow security and increase self-confidence, which reflects in better quality of care provided^(17)^.

In the period after the training, the reduction of acquired competences may occur. The period of one year or less is already recognized in several studies as the one with the greatest decline in retention^(10,13,14)^. A cohort evaluating the theoretical and practical knowledge of nurses in CPR six months after an educational intervention identified a reduction in theoretical knowledge of 14.5%; in the practical test the percentage of correct answers immediately after training was 79.5%, reducing to 51.5% in the following six months^(18)^.

The importance of training frequency is consensus as a way to reduce the loss of these skills over time. A systematic review of the literature reiterates this information by demonstrating that any prior training is associated with better skills, compared to no prior training, due to a cumulative effect of knowledge that is generated after each intervention^(19)^. Therefore, the more training and qualification, the better the professional performance.

A randomized clinical trial with nurses from a hospital in Canada showed that those who trained more and with a shorter time interval between training had better practical performance in CPR, as 58% of those trained monthly over a 12-month period performed CPR better than those in all other groups (only 26% in the group that trained every 3 months performed CPR satisfactorily, p = 0.008; 21% in the group that trained every 6 months, p = 0.002 and 15% in the group of 12 months, p < 0.001)^(20)^. In nursing students, research showed that performance improved at all training intervals evaluated (daily, weekly, monthly and quarterly), although shorter intervals resulted in a faster rate of improvement for general compression and ventilation skills^(17)^.

Health institutions, especially large ones, have difficulty in carrying out continuing education processes and developing training, especially with a very short time interval. Despite this, for health professionals, with greater exposure to CPA victims and the need for a higher percentage of skills retention, around 80%, more frequent training with intervals of up to 12 months is indicated, considering that after this period the skills can already reduce to values close to 70%^(21)^.

The maintenance of these optimal skills in resuscitation is related to a multitude of factors such as the type of training, frequency, methodologies adopted, personal and professional profile, among others. Training applied in another study significantly improved only the test results immediately after training and at three months, which did not occur in the following six months, where the nurses' scores returned to values close to those before the intervention, indicating the need for other educational activity earlier^(22)^.

Regarding the training strategy used in continuing education actions on this subject, studies have been developed seeking to identify which training method can favor greater learning and retention of CPR skills and suggest that training in high-fidelity simulation is superior to training on CPR low-fidelity mannikins^(23,24)^. Despite this, the theoretical and practical training using the low-fidelity simulation adopted in this study improved and helped to retain the professionals' knowledge and skills, and this is especially important for institutions with more limited resources, which also need to train their staff.

Another aspect is that a short-term training program, with low-technology materials, carried out in-service and with more spaced intervals between training, such as the one applied in this study, can also facilitate its replication in other hospital institutions. A study using short-term (1 h 30 min) theoretical and practical CPR teaching, using a low-cost model, was also effective in training health professionals, including people with medium to high schooling^(25)^.

It is believed that the combination of theoretical and practical training and the limited number of people per training period contributed to the optimization of results and less loss of practical skills. The most effective method for retaining CPR knowledge and skills and the appropriate time frame are not yet known, but evidence suggests that brief, frequent, repetitive, or deliberate practice used in collaboration with low- or high- fidelity simulation can be a potential strategy to improve nurse retention over time^(26)^.

It is known that continuing education in the work environ-ment has its challenges, but it contributes to greater safety in care and to professionals in-depth knowledge, which reflects in better quality in patient care^(27)^. A significantly higher survival rate among people who suffered cardiac arrest was achieved after BLS training of hospital nurses in India (52.9% post-training versus 27.5% pre-training)^(28)^. There is also a positive association between participants' performance in CPR, frequency of training, and whether CPR training was provided in the workplace^(29)^.

The limitations of this study are related to its performance in a single center, which may not represent other realities. The lack of a control group led the same group to answer assessment instruments repeatedly, although this was minimized through changes in data collection instruments that masked the tests to the participant. Another aspect is that it was performed in a simulated environment, instead of observing nurses and nursing technicians performing CPR in a real-life situation. Thus, we could not measure the effect of the interventions on daily clinical practice. Despite this, the results presented provide subsidies for planning health services in relation to the education of nursing professionals in their own workplace and guide training and qualification in CPR.

## CONCLUSION

The development of this study brought important elements about the in-service continuing education of nursing professionals in CPR. The interventions carried out were adequate to improve the professionals' knowledge and skills and the retention of skills acquired during a period of nine months without training, were greater after retraining, which reinforces the importance of continuity of actions.

Ensuring the retention of CPR skills is challenging, both for professionals and for health services; however, having specific training strategies, maintaining regularity and a defined interval between training are fundamental. We showed that training over a period of nine months, even using a low-cost and short-term model, was effective in retaining knowledge and skills.

Finally, we hope that this study can support health institu-tions regarding the planning of actions aimed at the professional development of nursing, in view of the relevant attribution of this category in CPA care, and serve as a reference for further studies to be carried out to also propose other feasible ways of in-service education.

## ASSOCIATE EDITOR

Thereza Maria Magalhães Moreira
